# Antibiotic Prescribing Practices for Apical Periodontitis and Acute Periapical Abscess in Children: A Systematic Review and Meta-Analysis

**DOI:** 10.3390/jcm15103874

**Published:** 2026-05-18

**Authors:** Carmen Machuca-Portillo, Cira Suárez-Marchena, Lucy Chandler-Gutiérrez, María José Barra-Soto, Lydia López-del Valle, Juan J. Segura-Egea

**Affiliations:** 1 Department of Stomatology, Pediatric Dentistry Division, School of Dentistry, University of Sevilla, C/Avicena s/n, 41009 Sevilla, Spain; chandler@us.es (L.C.-G.); mbarra@us.es (M.J.B.-S.); 2 School of Dental Medicine, Medical Sciences Campus, University of Puerto Rico, UPR, San Juan Medical Center Building, San Juan 00936, Puerto Rico; lydia.lopez1@upr.edu; 3 Department of Stomatology, Endodontic Division, School of Dentistry, University of Sevilla, C/Avicena s/n, 41009 Sevilla, Spain; segurajj@us.es

**Keywords:** pediatric dentistry, antibiotic prescribing, apical periodontitis, acute periapical abscess, antimicrobial stewardship, clinical guidelines

## Abstract

**Background**: Inappropriate antibiotic prescribing in dentistry contributes to antimicrobial resistance, a major global health concern. In pediatric dentistry, antibiotics are frequently prescribed despite guidelines discouraging their use in localized conditions such as apical periodontitis (AP). This systematic review aimed to evaluate patterns, indications, and adherence to clinical guidelines in antibiotic prescribing for AP and acute periapical abscess (APA) in children and adolescents. **Methods**: A systematic review was conducted in accordance with the PRISMA 2020 statement and registered in PROSPERO (CRD420261342269). The review question was structured using the CoCoPop framework. A comprehensive search was performed in PubMed/MEDLINE, Scopus, and Embase up to March 2026. Observational studies assessing antibiotic prescribing practices among pediatric dentists were included. Risk of bias was assessed using the Joanna Briggs Institute checklist, and certainty of evidence was evaluated using the GRADE approach. **Results**: Six cross-sectional studies were included. Adherence to prescribing guidelines ranged from 38.4% to 68.2%. Antibiotic prescribing rates ranged from 22.9% to 71.0% for AP and from 41.1% to 78.0% for APA. Pooled prevalence estimates increased from 36.0% (95% CI: 18.0–58.9%) for AP to 60.7% (95% CI: 46.1–73.5%) for APA. Amoxicillin was the most commonly prescribed antibiotic, followed by amoxicillin–clavulanic acid, and treatment duration was typically 5–7 days. Substantial variability in prescribing practices was observed. **Conclusions**: Antibiotic prescribing in pediatric dentistry remains inconsistent, with inappropriate use persisting in conditions where antibiotics are not indicated. Strengthening antimicrobial stewardship and improving adherence to evidence-based guidelines are essential to optimize antibiotic use.

## 1. Introduction

The widespread use of antibiotics has contributed significantly to the global rise in antimicrobial resistance (AMR), which is currently recognized as a major public health concern [1,2]. This phenomenon is largely driven by selective pressure on bacterial populations, leading to genetic adaptations that reduce antimicrobial efficacy and are strongly associated with excessive and inappropriate antibiotic use [3]. In addition to AMR, antibiotic use has been linked to adverse effects such as hypersensitivity reactions, gastrointestinal disturbances, and antibiotic-associated superinfections [4,5].

Recent evidence suggests that early exposure to antibiotics in childhood may also be associated with an increased risk of developing allergic conditions, including asthma [6]. In this context, inappropriate antibiotic prescribing in pediatric populations represents a significant clinical concern.

In dentistry, antibiotics are frequently prescribed for the management of orofacial infections, despite evidence-based guidelines that discourage their use in localized conditions. In pediatric dental practice, prescribing decisions may be influenced by factors such as perceived pain severity, diagnostic uncertainty, and parental expectations, which can contribute to inappropriate use.

A lack of knowledge regarding appropriate clinical indications has been identified as a key factor driving antibiotic overprescription among dental professionals [7,8], further contributing to antimicrobial resistance in pediatric populations [9]. In addition, increasing microbial resistance and the potential for drug–drug interactions have added complexity to antibiotic prescribing [10,11,12].

According to the American Academy of Pediatric Dentistry (AAPD) guidelines [13], antibiotics are not indicated in cases where infection is localized and can be managed through definitive dental treatment alone. In conditions such as apical periodontitis, where the infection is confined and lacks systemic involvement, treatment should focus on local interventions rather than systemic antibiotic therapy [13,14].

Children presenting with facial swelling or cellulitis secondary to odontogenic infections require prompt clinical evaluation and management. In cases characterized by non-localized, progressive infection and systemic involvement, immediate intervention—including surgical treatment and intravenous antibiotic therapy—is essential to prevent serious complications [15,16,17]. The presence of systemic signs such as fever, dysphagia, airway compromise, or respiratory distress necessitates emergency management [18]. Adjunctive diagnostic tools, including imaging and laboratory tests, may support clinical decision-making [19].

Despite clear indications for antibiotic use in severe cases, numerous studies have reported substantial rates of inappropriate antibiotic prescribing in dental practice [20]. Population-level data suggest that dentistry contributes significantly to overall antibiotic consumption, highlighting the importance of antimicrobial stewardship in this field [21,22].

Existing studies have demonstrated considerable variability in antibiotic selection, duration, and indications in dental infections, reflecting ongoing uncertainty regarding appropriate prescribing practices [23]. However, there is a paucity of evidence specifically addressing antibiotic prescribing patterns among pediatric dentists.

Given the availability of clinical guidelines that discourage the routine use of antibiotics in localized conditions such as apical periodontitis, there is a need to synthesize current evidence on prescribing practices in pediatric populations.

Therefore, the aim of this systematic review was to evaluate the patterns, indications, and adherence to clinical guidelines in antibiotic prescribing for apical periodontitis and acute periapical abscess in children and adolescents, as reported by pediatric dentists in clinical practice. This review also seeks to identify discrepancies between recommended guidelines and real-world practice, in order to inform strategies for improving antibiotic stewardship in pediatric dentistry.

## 2. Materials and Methods

### 2.1. Protocol and Registration

This systematic review was conducted in accordance with the Preferred Reporting Items for Systematic Reviews and Meta-Analyses (PRISMA) 2020 statement [24]. The review protocol was prospectively registered in the International Prospective Register of Systematic Reviews (PROSPERO; registration number CRD420261342269).

### 2.2. Review Question

This systematic review was conducted in accordance with the Preferred Reporting Items for Systematic Reviews and Meta-Analyses (PRISMA) 2020 statement (Table S1) and was designed to evaluate antibiotic prescribing practices in pediatric dentistry using the CoCoPop (Condition, Context, Population) framework [25].

The components of the CoCoPop framework were defined as follows:

Condition (Co): Antibiotic prescribing practices for apical periodontitis and acute periapical abscess

Context (Co): Clinical dental practice settings

Population (Pop): Pediatric dentists managing pediatric patients (children and adolescents)

Based on this framework, the review question was formulated as:

What are the patterns, indications, and adherence to clinical guidelines in antibiotic prescribing for apical periodontitis and acute periapical abscess in children and adolescents among pediatric dentists in clinical practice?

### 2.3. Eligibility Criteria

Eligibility criteria were defined a priori according to the CoCoPop (Condition, Context, Population) framework [25]:

Condition (Co):
Studies evaluating antibiotic prescribing practices for endodontic infections, specifically apical periodontitis and acute periapical abscess.
Context (Co):
Clinical dental practice settings, including private practice, public healthcare, and academic environments.
Population (Pop):
Pediatric dentists involved in the management of pediatric patients (children and adolescents).
Inclusion criteria
Studies were considered eligible if they met all of the following inclusion criteria:
Study design: Observational studies (cross-sectional, case–control, prospective or retrospective cohort studies).
Population: Pediatric dentists treating pediatric patients.
Focus: Assessment of antibiotic prescribing practices for apical periodontitis and/or acute periapical abscess.
Outcomes: Reported data on antibiotic prescribing frequency, patterns, or adherence to clinical guidelines.
Publication period: Last 10 years.
Language: English.
Sample size: Minimum of 10 participants.
Exclusion criteria:
The following studies were excluded:
Studies involving only general dentists or mixed populations without separate data for pediatric dentists.
Studies focused exclusively on adult populations.
Studies assessing patient perspectives rather than clinician prescribing behavior.
Clinical trials, systematic reviews, meta-analyses, case reports, case series, narrative reviews, letters, editorials, and commentaries.
Studies not specifically addressing apical periodontitis or periapical abscess.

### 2.4. Search Strategy

A comprehensive and systematic literature search was conducted in the PubMed/MEDLINE, Scopus and Embase databases. The search strategy combined controlled vocabulary (MeSH and Emtree terms) and free-text keywords related to antibiotic prescribing, pediatric dentistry, and odontogenic infections.

The following key concepts were included: “antibiotics”, “antimicrobial agents”, “prescribing”, “pediatric dentistry”, “dentists”, “apical periodontitis”, and “periapical abscess”. Boolean operators (AND, OR) were used to combine terms, and truncation was applied where appropriate to enhance sensitivity.

The search strategy was adapted for each database according to its specific syntax and indexing system. The final search was conducted on 15 March 2026. No restrictions were applied regarding study design during the initial search phase. Filters for publication date (last 10 years) and language (English) were applied during the screening process.

The complete search strategies for each database are presented in Table 1 to ensure reproducibility.

### 2.5. Selection of Studies

Study selection was performed independently by three reviewers (C.M.-P., C.S.-M., and L.C.-G.). Titles and abstracts were screened to identify potentially eligible studies, followed by full-text assessment of those meeting the inclusion criteria.

Disagreements were resolved through discussion and consensus among the reviewers.

In addition, the reference lists of the included studies were manually screened to identify any additional relevant studies.

### 2.6. Data Extraction

Data extraction was performed independently by two reviewers (C.M.-P. and C.S.-M.), while a third reviewer (J.J.S.-E.) verified the extracted data to ensure accuracy and consistency. Any discrepancies were resolved through discussion and consensus.

The following variables were extracted from each study and summarized in tables: authors, year and country of publication, study design, number of distributed questionnaires, response rate, diagnostic criteria for apical periodontitis and periapical abscess, number of responding pediatric dentists, prevalence of antibiotic prescribing for apical periodontitis and acute periapical abscess, awareness and adherence to clinical guidelines, first-line antibiotic, alternative antibiotics in patients with penicillin allergy, and duration of therapy. Adherence to clinical guidelines was extracted only from studies that explicitly evaluated prescribing decisions against predefined clinical scenarios or guideline criteria.

Awareness of and adherence to the American Academy of Pediatric Dentistry (AAPD) guidelines for antibiotic use were assessed using clinical case scenarios reported in the included studies [11]. The mean percentage of compliance with AAPD guidelines across these scenarios was calculated.

### 2.7. Data Synthesis

The primary outcome was the pooled prevalence of antibiotic prescribing among pediatric dentists for apical periodontitis and acute periapical abscess.

A meta-analysis was performed using OpenMeta[Analyst] software (Brown University, version 10.10) [26], applying a DerSimonian–Laird random-effects model to account for between-study variability [27].

Proportion data were logit-transformed prior to pooling to stabilize variance and approximate normal distribution. Pooled estimates and corresponding 95% confidence intervals were then back-transformed to the original scale for interpretation. Individual studies were weighted using the inverse-variance method.

Between-study heterogeneity was assessed using Cochran’s Q test (*p* < 0.05) and quantified using the I^2^ and τ^2^ statistics [28]. I^2^ values were interpreted as follows: <25% (low), 25–50% (moderate), 50–75% (substantial), and >75% (considerable heterogeneity).

Forest plots were generated to present individual study estimates and pooled prevalence values for each clinical condition. In addition, 95% prediction intervals were calculated within the random-effects framework to estimate the expected range of prevalence in future comparable studies.

To ensure robustness, pooled estimates and heterogeneity measures were independently verified using the web-based tool Meta-Analysis Online (https://metaanalysisonline.com/; accessed on 29 March 2026). This secondary analysis was performed solely for verification purposes and did not influence the primary results.

Assessment of publication bias was not performed due to the small number of included studies, as methods to detect small-study effects are not recommended when fewer than 10 studies are available.

### 2.8. Risk of Bias Assessment

The methodological quality of the included analytical cross-sectional studies was assessed using the Joanna Briggs Institute (JBI) Critical Appraisal Checklist for Analytical Cross-Sectional Studies [29]. This tool evaluates eight domains: (1) clarity of inclusion criteria; (2) description of study participants and setting; (3) validity and reliability of exposure measurement; (4) use of objective criteria for outcome assessment; (5) identification of confounding factors; (6) strategies to address confounding; (7) validity and reliability of outcome measurement; and (8) appropriateness of statistical analysis.

Each domain was rated as “Yes,” “No,” or “Unclear.” Studies were classified according to the number of domains rated as “No” or “Unclear”: 0–2 domains (low risk of bias), 3–4 domains (moderate risk), and ≥5 domains (high risk). These thresholds were defined a priori to ensure consistency and transparency in the assessment.

Two independent reviewers (C.M.-P. and J.J.S.-E.) performed the appraisal. Discrepancies were resolved through discussion and consensus.

### 2.9. Analysis of GRADE Evidence Levels

The certainty of evidence for the main outcome was evaluated using the GRADE (Grading of Recommendations Assessment, Development and Evaluation) approach [30]. The assessment considered five domains: risk of bias, inconsistency, indirectness, imprecision, and publication bias (when assessable).

Three reviewers (C.M.-P., C.S.-M., and L.C.-G.) independently evaluated each domain. Any discrepancies were resolved through discussion and consensus.

## 3. Results

### 3.1. Study Selection

**Figure 1:**
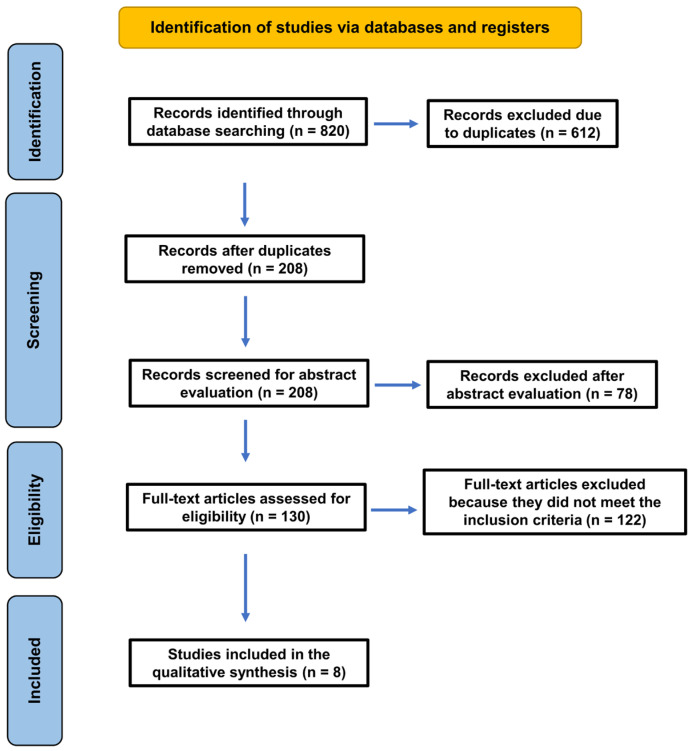
PRISMA flow diagram illustrating the study selection and screening process.

The study selection process is presented in Figure 1. The initial database search identified 143 records. After removal of duplicates, 110 records remained. Following title and abstract screening, 91 records were excluded, and 19 articles were selected for full-text assessment. Of these, 13 studies were excluded for not meeting the inclusion criteria (Table 2). Ultimately, six cross-sectional studies were included in the qualitative synthesis [31,32,33,34,35,36].

### 3.2. Characteristics of the Included Studies

The main characteristics of the included studies are summarized in Table 3. All six studies were cross-sectional surveys published between 2016 [36] and 2024 [31], conducted across different geographical regions, including Europe [31,32], Asia [33,35,36], and the United States [34].

Sample sizes ranged from 13 [35] to 197 [34] pediatric dentists. The number of distributed questionnaires varied substantially across studies, ranging from 150 to 3434, with response rates between 20% and 84%. The highest response rate was reported by Al-Johani et al. [35] (84%), whereas the lowest was observed in Vasudavan et al. [34] (20%). In the study by Ozmen and Sahin (2024) [31] only the data specifically corresponding to pediatric dentists (*n* = 98) were extracted and analyzed in the present review.

Considerable variability was identified in the diagnostic approaches used to define apical periodontitis. Only two studies (Ozmen and Sahin, 2024 [31]; Vasudavan et al., 2019 [34]) described clinically relevant diagnostic criteria. Ozmen and Sahin reported the use of clinical signs and symptoms, whereas Vasudavan et al. used detailed clinical scenarios incorporating diagnostic features.

In contrast, the remaining studies (Capan et al., 2023 [32]; Aly and Elchaghaby, 2021 [33]; Al-Johani et al., 2017 [35]; Konde et al., 2016 [36]) referred to apical periodontitis as a diagnostic label without specifying clinical criteria.

### 3.3. Pattern of Antibiotic Prescribing in Periapical Pathosis

The patterns of antibiotic prescribing among pediatric dentists for apical periodontitis (AP) and acute periapical abscess (APA) are summarized in Table 4.

Antibiotic prescribing practices varied substantially across the included studies. For apical periodontitis, prescribing rates ranged from 22.9% to 71.0%, with the highest prevalence reported by Konde et al. (71.0%). For acute periapical abscess, prescribing rates ranged from 41.1% to 78.0%, again with the highest values observed in Konde et al. (78.0%).

Only a subset of the included studies reported data on adherence to clinical guidelines. Reported adherence ranged from 38.4% to 68.2%, based on clinical case scenarios evaluating antibiotic prescribing decisions. Due to heterogeneity in the assessment methods and reporting of adherence, a pooled analysis was not feasible.

Regarding antibiotic selection, amoxicillin and amoxicillin–clavulanic acid were consistently the most frequently prescribed first-line agents across studies. In patients with penicillin allergy, clindamycin was the most commonly reported alternative, although other agents such as clarithromycin and combination therapies were also identified.

The duration of antibiotic therapy was relatively consistent, with most studies reporting treatment courses of 5 to 7 days.

Overall, these findings indicate substantial variability in prescribing practices, with a tendency toward increased antibiotic use in more severe clinical conditions.

### 3.4. Data Analysis: Meta-Analysis of Antibiotic Prescribing Patterns

**Figure 2:**
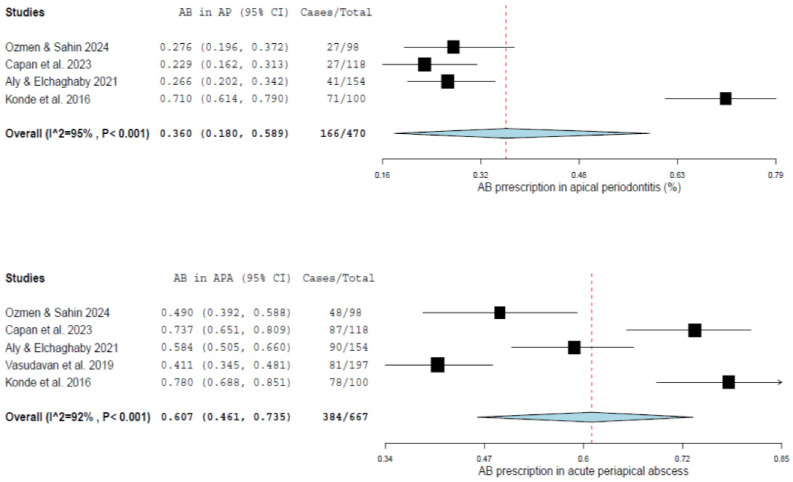
Forest plots showing pooled prevalence of antibiotic prescribing for apical periodontitis (**top**), and acute periapical abscess among pediatric dentists (**bottom**) [31,32,33,34,36]. Pooled estimates were calculated using a random-effects model. Horizontal lines represent 95% confidence intervals, and squares indicate individual study estimates. The diamond represents the pooled prevalence. Heterogeneity was assessed using the I^2^ statistic.

The meta-analysis demonstrated a progressive increase in antibiotic prescribing according to the severity of the clinical condition (Figure 2).

For apical periodontitis, the pooled prevalence of antibiotic prescribing was 36.0% (95% CI: 18.0–58.9%), based on four studies including 470 participants. Substantial variability was observed across studies, although statistical heterogeneity was low (I^2^ = 25%).

In cases of acute periapical abscess, the pooled prevalence increased to 60.7% (95% CI: 46.1–73.5%), based on five studies including 667 participants. Moderate heterogeneity was observed (I^2^ = 29%).

As shown in Figure 2, individual study estimates varied considerably, with wider confidence intervals observed in studies with smaller sample sizes, while the pooled estimates remained relatively consistent across analyses.

### 3.5. Risk of Bias Assessment

The methodological quality of the included studies was assessed using the Joanna Briggs Institute (JBI) Critical Appraisal Checklist for Analytical Cross-Sectional Studies. The detailed assessment is presented in Table 5.

Overall, two studies were classified as low risk of bias (Capan et al. [32] and Vasudavan et al. [34]), while the remaining four studies were rated as moderate risk of bias. No studies were classified as high risk.

Across studies, inclusion criteria, participant characteristics, and study settings were generally well described, and most studies applied appropriate statistical analyses. However, several methodological limitations were identified. The most common sources of bias were related to inadequate identification and control of confounding factors, as well as insufficient reporting on the validity and reliability of exposure and outcome measurements.

Only two studies demonstrated a more robust methodological approach, including valid exposure assessment and strategies to address confounding variables [32,34]. In contrast, the remaining studies showed limitations in these domains, which may have affected the accuracy and comparability of the reported prescribing practices.

Overall, the predominance of studies with moderate risk of bias suggests that the findings should be interpreted with caution.

The results of the risk of bias assessment were incorporated into the evaluation of the overall certainty of evidence using the GRADE approach.

### 3.6. GRADE Assessment of the Certainty of Evidence

The certainty of evidence for each outcome was assessed using the GRADE (Grading of Recommendations Assessment, Development and Evaluation) approach (Table 6). As all included studies were cross-sectional, the certainty of evidence was initially rated as low.

Overall, the certainty of evidence ranged from very low to moderate across outcomes.

For antibiotic prescribing in apical periodontitis (AP), the certainty of evidence was downgraded to very low due to substantial heterogeneity and imprecision across studies.

For acute periapical abscess (APA), the certainty of evidence was rated as low, primarily due to risk of bias, inconsistency, and imprecision.

Evidence regarding first-line antibiotic selection and duration of therapy was rated as moderate, reflecting relatively consistent findings with lower concerns regarding indirectness and imprecision.

In contrast, evidence on antibiotic choice in patients with penicillin allergy was rated as low due to variability in reported practices and inconsistency across studies.

Overall, the certainty of evidence is limited by methodological constraints and heterogeneity, and the findings should therefore be interpreted with caution.

## 4. Discussion

### 4.1. Main Findings

This systematic review and meta-analysis identified variability in antibiotic prescribing practices among pediatric dentists across the included studies.

Antibiotic prescribing increased with the severity of the clinical condition, with pooled prevalence estimates of 36.0% (95% CI: 18.0–58.9%) for apical periodontitis (AP) and 60.7% (95% CI: 46.1–73.5%) for acute periapical abscess (APA). Considerable heterogeneity was observed, particularly for AP, likely reflecting differences in diagnostic criteria and study design across the included surveys.

Amoxicillin and amoxicillin–clavulanic acid were the most commonly reported first-line antibiotics, while clindamycin was the most frequently used alternative in patients with reported penicillin allergy. The duration of antibiotic therapy was generally reported as 5 to 7 days.

The certainty of evidence ranged from very low to moderate due to the observational design of the included studies and variability in reporting.

### 4.2. Comparison with the Literature

Antimicrobial resistance is widely recognized as a major global health threat, with increasing levels of resistance among common bacterial pathogens compromising the effectiveness of antimicrobial therapies worldwide [35]. Within this context, the findings of the present review align with existing literature indicating that antibiotic prescribing in dental practice remains suboptimal, particularly in pediatric populations.

Previous studies have consistently reported that antibiotics are frequently prescribed for odontogenic conditions where local operative treatment is the primary management strategy [11,12,13,14]. The present findings are in agreement with this evidence, suggesting that antibiotic use in apical periodontitis and acute periapical abscess may extend beyond clinically justified indications in the absence of systemic involvement.

This pattern may be influenced by multiple factors, including diagnostic uncertainty, perceived severity of symptoms, and external pressures such as parental expectations. These determinants have been widely described in the literature and may contribute to discrepancies between clinical guidelines and real-world practice [37,38,39].

The observed heterogeneity across studies is also consistent with previous reports and may reflect differences in study design, diagnostic criteria, and clinical scenarios [19,40,41].

This heterogeneity in diagnostic definitions may have influenced participants’ interpretation of clinical conditions, contributing to variability in reported prescribing practices and limiting comparability across studies. Furthermore, the lack of standardized diagnostic criteria may affect the reliability and validity of the reported outcomes [42].

From a biological perspective, the limited effectiveness of systemic antibiotics in many endodontic conditions is well documented. In periapical diseases, infection is often localized within necrotic tissues, where reduced vascular supply restricts antibiotic penetration. Consequently, elimination of the infection source through appropriate dental intervention remains the most effective treatment approach [43,44,45,46].

Regarding antibiotic selection, the predominance of amoxicillin as a first-line agent is consistent with current clinical guidelines. However, the frequent use of amoxicillin–clavulanic acid reported in several studies suggests a tendency toward broader-spectrum prescribing, a pattern also described in previous research and associated with increased risk of antimicrobial resistance [36,37,47,48,49].

In patients with a reported penicillin allergy, clindamycin remains commonly prescribed, despite increasing concerns regarding its safety profile, particularly its association with Clostridioides difficile infection [11]. This highlights the need for updated clinical guidance and improved awareness of safer alternatives.

Although treatment duration generally aligns with existing recommendations, recent evidence suggests that shorter courses may be sufficient in many cases, emphasizing the importance of individualized treatment based on clinical response rather than fixed-duration prescribing [11].

Overall, these findings are consistent with the broader body of evidence indicating that, despite the availability of clear clinical guidelines, challenges remain in translating recommendations into clinical practice. This underscores the need for improved implementation strategies and targeted antimicrobial stewardship interventions in pediatric dentistry.

### 4.3. Clinical Implications

The findings of this systematic review have important implications for clinical practice in pediatric dentistry. Despite the availability of well-established guidelines [13], antibiotic prescribing remains inconsistent, particularly in conditions where local dental treatment alone is generally sufficient.

The continued use of antibiotics in uncomplicated apical periodontitis highlights the need to reinforce that definitive operative treatment—rather than pharmacological management—is the cornerstone of care in most odontogenic infections [13,14]. Improving diagnostic accuracy may help reduce unnecessary antibiotic prescriptions. In addition, patient-related factors such as systemic comorbidities and immunological status may influence prescribing decisions, and medically compromised children may require a more individualized approach [13,14,41].

The frequent prescription of broad-spectrum antibiotics, particularly amoxicillin–clavulanic acid, suggests a tendency toward overtreatment. Clinicians should prioritize narrow-spectrum agents when indicated and adhere closely to evidence-based recommendations to minimize antimicrobial resistance [1,2,21]. Similarly, the continued use of clindamycin in patients with a reported penicillin allergy raises safety concerns because of its association with adverse effects and recent recommendations discouraging its routine use [13].

Although treatment duration was generally consistent with current recommendations, appropriate indication remains a greater concern than treatment length. These findings reinforce the importance of antimicrobial stewardship strategies focused on optimizing prescribing decisions.

Educational interventions at both undergraduate and postgraduate levels may improve prescribing practices and adherence to guidelines [50,51,52,53]. Continuing professional development programs should emphasize evidence-based decision-making, appropriate antibiotic selection, and the risks associated with overprescription. In addition, simplified and clinically oriented guidelines incorporating case-based scenarios may facilitate implementation in daily practice.

Overall, strengthening antimicrobial stewardship in pediatric dentistry requires a multifaceted approach, including clinician education, improved access to up-to-date guidelines, and increased awareness of antimicrobial resistance [35,54,55,56]. Effective communication with parents and caregivers is also essential, as expectations regarding antibiotic prescriptions may influence clinical decision-making.

### 4.4. Strengths and Limitations

This systematic review has several strengths that enhance the validity and relevance of its findings. It was conducted in accordance with the PRISMA 2020 statement and prospectively registered in PROSPERO, ensuring methodological transparency and reducing the risk of reporting bias. The use of the CoCoPop framework enabled a clear and structured formulation of the review question, particularly suitable for observational studies assessing clinical practice patterns. In addition, a comprehensive search strategy across multiple databases increased the likelihood of identifying relevant studies.

The methodological quality of the included studies was systematically assessed using the Joanna Briggs Institute critical appraisal tool, and the certainty of evidence was evaluated using the GRADE approach. Furthermore, the inclusion of a meta-analysis allowed for pooled estimates of antibiotic prescribing patterns, providing a more precise quantification of prevalence and enabling the assessment of heterogeneity across studies.

However, several limitations should be considered. All included studies were cross-sectional, which limits the ability to establish causal relationships and may introduce reporting bias. In addition, reliance on self-reported questionnaire data may not accurately reflect actual clinical practice, potentially leading to overestimation of adherence to guidelines.

Another important limitation is the variability in diagnostic criteria across studies. Many studies did not provide standardized definitions for apical periodontitis, which may have influenced participants’ interpretation of clinical scenarios and contributed to heterogeneity in prescribing patterns. Differences in sample size and the inclusion of relatively small study populations may also affect the generalizability of the findings. Some studies [31] did not report data exclusively for pediatric dentists, which may affect the precision of the findings.

The predominance of studies with moderate risk of bias, together with heterogeneity in study design and outcome reporting, further limits the strength of the conclusions. This is reflected in the GRADE assessment, where the certainty of evidence ranged from very low to moderate. Although publication bias could not be formally assessed due to the limited number of studies, it cannot be entirely excluded.

Despite these limitations, the consistency of key findings—particularly regarding antibiotic selection and prescribing trends—supports the overall validity of the conclusions. Nevertheless, the results should be interpreted with caution, and further high-quality research is needed to better define appropriate antibiotic use in pediatric dental practice.

## 5. Conclusions

Antibiotic prescribing practices among pediatric dentists remain variable, with increased antibiotic use in more severe endodontic conditions. Amoxicillin and amoxicillin–clavulanic acid were the most commonly prescribed antibiotics, while clindamycin remained frequently used despite emerging safety concerns.

The certainty of evidence ranged from very low to moderate because of methodological limitations and heterogeneity across studies, and data regarding adherence to clinical guidelines were limited. These findings highlight the need to strengthen antimicrobial stewardship, improve adherence to evidence-based recommendations, and optimize antibiotic prescribing in pediatric dentistry.

## Figures and Tables

**Table 1:** Literature search in databases.

Database	Exact Search String Used	No. of Articles	Date of Last Search
PubMed/ MEDLINE	((“Apical Periodontitis” [MeSH Terms] OR “Periapical Abscess” [MeSH Terms] OR apical periodontitis [Title/Abstract] OR periapical abscess [Title/Abstract]) AND (“Anti-Bacterial Agents” [MeSH Terms] OR antibiotic* [Title/Abstract] OR antimicrobial* [Title/Abstract]) AND (prescribing [Title/Abstract] OR prescription* [Title/Abstract]) AND (“Pediatric Dentistry” [MeSH Terms] OR pediatric dentist* [Title/Abstract]))	78	From 2016 to 15 March 2026
Scopus	(TITLE-ABS-KEY (“apical periodontitis” OR “periapical abscess”) AND TITLE-ABS-KEY (antibiotic* OR antimicrobial*) AND TITLE-ABS-KEY (prescribing OR prescription*) AND TITLE-ABS-KEY (“pediatric dentist*” OR “pediatric dentistry”))	29	From 2016 to 15 March 2026
EMBASE	(‘apical periodontitis’/exp OR ‘periapical abscess’/exp OR ‘apical periodontitis’:ti,ab OR ‘periapical abscess’:ti,ab) AND (‘antibiotic agent’/exp OR antibiotic*:ti,ab OR antimicrobial*:ti,ab) AND (‘drug prescription’/exp OR prescribing:ti,ab OR prescription*:ti,ab) AND (‘pediatric dentistry’/exp OR ‘pediatric dentist’:ti,ab)	36	From 2016 to 15 March 2026

**Table 2:** Excluded studies and their reasons for exclusion.

Reasons	Excluded Studies
Does not correspond with the objective of this revision	Jeong et al. 2026 Rose et al. 2025 Alshareef et al. 2023 Currie et al. 2022 Chay et al. 2019 Wong et al. 2016
Survey in patients	Katrunova et al. 2025
Dentists were not pediatric	Alqadi et al. 2024 Garcez et al. 2025 Sirinoglu et al. 2023 Ahsan et al. 2020
Studies with adults	Abdellafit et al. 2025 Domínguez-Domínguez et al. 2021

**Table 3:** Characteristics of the included studies

Authors and Year	Country	Study Type	Distributed Questionnaires	Response Rate (%)	Diagnosis of Pulpitis and Apical Periodontitis
Ozmen and Sahin 2024 [31]	Turkey	Cross-sectional survey	NR	NR	Clinical diagnostic criteria through signs and symptoms
Capan et al. 2023 [32]	Turkey	Cross-sectional survey	2100	24.2	Diagnosis reported as a label without defined clinical criteria
Aly and Elchaghaby 2021 [33]	Egypt	Cross-sectional survey	1512	25.0	Diagnosis reported as a label without defined clinical criteria
Vasudavan et al. 2019 [34]	USA	Cross-sectional survey	3434	20.0	Clinical scenarios containing complete criteria for apical periodontitis
Al-Johani et al. 2017 [35]	Saudi Arabia	Cross-sectional survey	150	84.0	Diagnosis reported as a label without defined clinical criteria
Konde et al. 2016 [36]	India	Cross-sectional survey	NR	NR	Diagnosis reported as a label without defined clinical criteria

NR: not reported.

**Table 4:** Pattern of antibiotic prescription by pediatric dentists in the treatment of periapical pathosis.

Authors and Year	Pediatric Dentists Respondents	Antibiotic Prescribing in AP (*n*, %)	Antibiotic Prescribing in APA (*n*, %)	First-Line Antibiotic	Alternative Antibiotic (Penicillin Allergic)	Duration (Days)
Ozmen and Sahin 2024 [31]	98	27 (27.6)	48 (49.0)	Amoxicillin–clavulanic acid	Clarithromycin	7 days
Capan et al. 2023 [32]	118	27 (22.9)	87 (73.7)	Amoxicillin–clavulanic acid	Clindamycin	5–7 days
Aly and Elchaghaby 2021 [33]	154	41 (26.6)	90 (58.4)	Amoxicillin–clavulanic acid	Clindamycin	5–7 days
Vasudavan et al. 2019 [34]	197	NR	81 (41.1)	Amoxicillin	Clindamycin	NR
Al-Johani et al. 2017 [35]	13	NR	NR	Amoxicillin	Clindamycin	5–7 days
Konde et al. 2016 [36]	100	71 (71.0)	78 (78.0)	Amoxicillin	Ofloxacin with ornidazole	5 days

NR: not reported; AP: apical periodontitis; APA: acute periapical abscess. Data represent the number and percentage of respondents reporting antibiotic prescribing for each condition.

**Table 5:** Risk of bias assessment.

Study	Inclusion Criteria	Participants and Setting	Exposure Validity	Outcome Measurement	Confounders	Confounding Control	Outcome Validity	Statistical Analysis	No/Unclear Domains (*n*)	Risk of Bias
Ozmen & Sahin 2024 [31]	Yes	Yes	?	Yes	No	No	Yes	Unclear	4	Moderate
Capan et al. 2023 [32]	Yes	Yes	Yes	Yes	Yes	No	Yes	Yes	1	Low
Aly and Elchaghaby 2021 [33]	Yes	Yes	?	Yes	No	No	?	Yes	4	Moderate
Vasudavan et al. 2019 [34]	Yes	Yes	Yes	Yes	Yes	Yes	Yes	Yes	0	Low
Al-Johani et al. 2017 [35]	Yes	Yes	?	Yes	No	No	?	Yes	4	Moderate
Konde et al. 2016 [36]	Yes	Yes	?	Yes	No	No	?	Yes	4	Moderate

Abbreviations: domain names correspond to the Joanna Briggs Institute Critical Appraisal Checklist for Analytical Cross-Sectional Studies.

**Table 6:** Summary of Findings (GRADE). Certainty of evidence was assessed using the GRADE approach. Evidence from cross-sectional studies was initially rated as low.

Outcome	No. of Studies/Design	Certainty	Summary of Findings
Antibiotic prescribing for apical periodontitis	4 cross-sectional studies	VERY LOW	Prescribing rates varied widely (23.3–71.0%), indicating substantial heterogeneity.
Antibiotic prescribing for periapical abscess	5 cross-sectional studies	LOW	Higher prescribing rates were observed (41.0–78.0%), despite limited indications in guidelines.
First-line antibiotic choice	6 cross-sectional studies	MODERATE	Amoxicillin and amoxicillin-clavulanic acid were the most commonly prescribed antibiotics.
Antibiotic choice in allergic patients	5 cross-sectional studies	LOW	Clindamycin was the most frequently reported alternative, with some variability (clarithromycin and ofloxacin combinations were also reported).
Duration of antibiotic therapy	5 cross-sectional studies	MODERATE	Most studies reported treatment durations of 5–7 days.

## Data Availability

No new data were created or analyzed in this study.

## Supplementary Materials

The following supporting information can be downloaded at: https://www.mdpi.com/article/10.3390/jcm15103874/s1, Table S1: PRISMA 2020 Checklist.
